# Exploring benzimidazole resistance in *Haemonchus contortus* by next generation sequencing and droplet digital PCR

**DOI:** 10.1016/j.ijpddr.2018.09.003

**Published:** 2018-09-19

**Authors:** Paulius Baltrušis, Peter Halvarsson, Johan Höglund

**Affiliations:** Swedish University of Agricultural Sciences, Department of Biomedical Sciences and Veterinary Public Health, Section for Parasitology, P.O. Box 7036, Uppsala, Sweden

**Keywords:** Strongyles, ddPCR, NGS-Sequencing, Benzimidazoles, Anthelmintic resistance

## Abstract

Anthelmintic resistance in gastrointestinal nematode (GIN) parasites of grazing ruminants is on the rise in countries across the world. *Haemonchus contortus* is one of most frequently encountered drug-resistant GINs in small ruminants. This blood-sucking abomasal nematode contributes to massive treatment costs and poses a serious threat to farm animal health. To prevent the establishment of resistant strains of this parasite, up-to-date molecular techniques need to be proposed which would allow for quick, cheap and accurate identification of individuals infected with resistant worms. The effort has been made in the previous decade, with the development of the pyrosequencing method to detect resistance-predicting alleles. Here we propose a novel droplet digital PCR (ddPCR) assay for rapid and precise identification of *H. contortus* strains as being resistant or susceptible to benzimidazole drugs based on the presence or absence of the most common resistance-conferring mutation F200Y (TAC) in the β tubulin isotype 1 gene. The newly developed ddPCR assay was first optimized and validated utilizing DNA templates from single-worm samples, which were previously sequenced using the next generation PacBio RSII Sequencing (NGS) platform. Subsequent NGS results for faecal larval cultures were then used as a reference to compare the obtained values for fractional abundances of the resistance-determining mutant allele between ddPCR and NGS techniques in each sample. Both methods managed to produce highly similar results and ddPCR proved to be a reliable tool which, when utilized at full capacity, can be used to create a powerful mutation detection and quantification assay.

## Highlights

• Sequencing results show country-wide distribution of resistant *H. contortus* in sheep.
• Two novel mutations for 198^th^ codon in β-tubulin were found in single-worm samples.
• Digital droplet PCR results correlated well with those derived from NGS sequencing.
• A new digital droplet PCR assay for mutation detection in *H. contortus* was created.

## Graphical abstract

**Figure:**
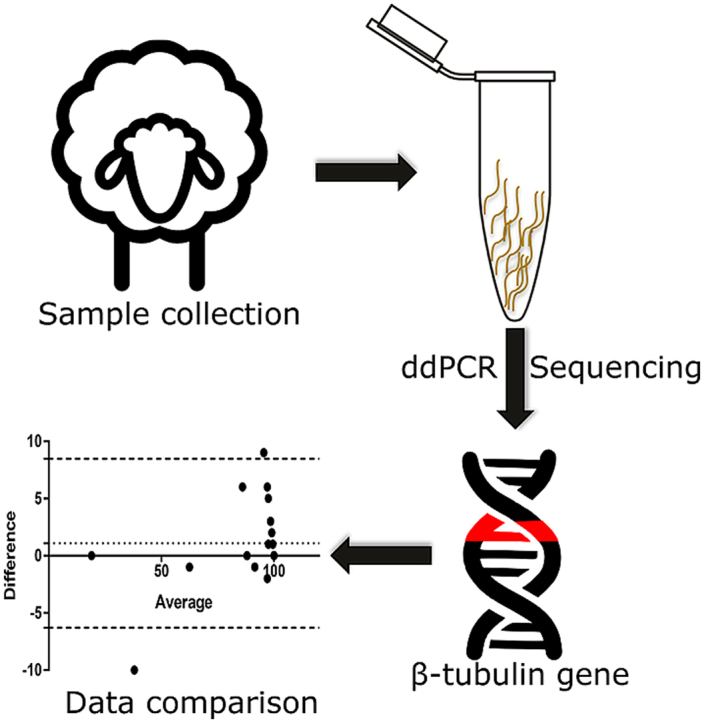

## 1 Introduction

The blood-sucking nematode of small ruminants *Haemonchus contortus* has become a thorn in the side for farmers across the world. Even though gastrointestinal nematode (GIN) infections in livestock are usually found to be mixed, *H. contortus* stands out as the most pathogenic and abundant species (Besier et al., 2016). In Australia, prevention costs against nematode parasites amount to the major part of the country's spending in the red meat industries (Lane et al., 2015). In the UK, total annual losses in the sheep sector due to GIN parasites constituted £84 million more than 10 years ago, and have since then likely increased (Nieuwhof and Bishop, 2005). In addition, developing countries, such as India, suffer from immense annual treatment costs which can be considerably detrimental to the countries' economies ($103 mln.) (McLeod, 2004). Apart from gross economic losses, impaired animal health and welfare are also key issues.

To date, three major drug classes are utilized to treat GIN infections in ruminants – benzimidazoles (BZ), levamisole (LEV), and macrocyclic lactones (ML), all of which are broad spectrum anthelmintics which reduce the existing worm burdens and if used persistently decrease pasture contamination and thus prevent the establishment of infective-stage larvae (L3) (Hoste et al., 2011; Sutherland and Leathwick, 2011). However, years of improper drug use have led to the development of anthelminthic resistance (AR) among these worms and somewhat halted the ever-increasing development of the animal production sector worldwide. Even in most European countries, such as the United Kingdom (Hong et al., 1996), Netherlands (Van den Brom et al., 2013), Spain (Requejo-Fernández et al., 1997), Switzerland (Schnyder et al., 2005), Germany (Bauer, 2001), Poland (Mickiewicz et al., 2017), Italy (Cringoli et al., 2007), France (Paraud et al., 2009), Denmark (Peña-Espinoza et al., 2014), Sweden (Höglund et al., 2009) and Norway (Domke et al., 2012), sheep and goat GIN parasite resistance towards benzimidazole anthelmintic drugs has become a common occurrence.

One of the most significant properties of *H. contortus*, is the propensity to develop resistance to anthelminthic drugs, which has been well documented and described by Kotze and Prichard (2016). This in part can be explained by very high gene flow between populations, remarkable within-population genetic diversity (Blouin et al., 1995; Troell et al., 2006) and high effective population size. This increased variability within-populations is especially true for β-tubulin genes, which account for resistance to BZ substance class of anthelmintic drugs (Beech et al., 1994).

To tackle the problem of growing resistance of *H. contortus* towards BZ drugs, alternatives to anthelminthic treatment must be considered and an end to ineffective drug use has to be implemented. To achieve this, a reliable, rapid and reasonably cost-effective screening-diagnostic tool is of paramount importance (Kenyon and Jackson, 2012). The current, gold standard for identifying clinical anthelmintic resistance in flocks of animals is the Faecal Egg Count Reduction Test (FECRT) (Coles et al., 2006). Albeit a well-described and firmly established method, it is lacking in sensitivity (Höglund et al., 2009). Furthermore, a number of elements can dramatically influence the outcome of the results, such as the level of excretion and aggregation of FEC, sample size and dilution factors, making this tool nothing more than a ‘rough estimation’ which is inefficient, difficult to interpret and reproduce (Levecke et al., 2012). Although a step towards developing and validating molecular tools for the detection, screening and evaluation of resistance towards anthelmintic drugs in parasitic nematodes was taken in the previous decade, mainly in the form of pyrosequencing (Von Samson-Himmelstjerna et al., 2007, 2009; Höglund et al., 2009), the search for novel, ‘better’ methods continues.

Droplet Digital PCR (ddPCR) is a recent modification of the third generation digital PCR. It boasts high accuracy, versatility and removes the need for standards or references in comparison to qPCR (Hindson et al., 2011). In this study, the ddPCR method was employed to create a universal protocol for the detection and quantification of the transversion occurring at the 200^th^ codon (P200) (T**T**C→T**A**C) in the β-tubulin isotype 1 gene, resulting in the acquisition of AR to BZ drugs in different strains of *H. contortus*. For this purpose, mixed ovine faecal larval culture samples containing *H. contortus*, derived from Swedish farms pre- and post-treatment with a BZ drug (albendazole), were subjected to molecular characterization of the β-tubulin isotype 1 gene locus, first by utilizing next generation PacBio RSII Sequencing (NGS) to obtain a reference read of the samples contents’ and then by the optimized ddPCR, developed by the authors. The fractional abundance (FA) values for both alleles (wild-type and P200 mutant) were compared between the two methods to determine if the newly developed protocol is reliable and precise. In addition to this, adult *H. contortus* single-worm samples, collected from 13 different countries were subjected to the same NGS of the β-tubulin isotype 1 gene locus and were further used not only in the initial development of the ddPCR protocol but also in the construction a Bayesian phylogenetic tree based on the retrieved exon sequences.

## 2 Material and methods

### 2.1 Sources of DNA

Genomic DNA was extracted from two different sources: 1) individual adult *H. contortus* worms and 2) mixed faecal larval cultures. The adult worms were previously isolated, bio-banked samples from naturally infected sheep (Troell et al., 2006), and were initially used in the ddPCR method development and optimisation stage. During the method validation stage, DNA samples, derived from a mixture of strongyles and extracted from faecal larval cultures collected from 13 sheep farms pre- and/or post-treatment around southern Sweden, were used (Supplementary Table 1) (Note: farms K-M only had a single pre- or post-treatment sample available). DNA extractions were performed according to the instructions of the supplier on thawed samples using NucleoSpin Tissue kit (Macherey Nagel).

### 2.2 DNA library creation and sequencing

Genomic DNA samples from *H. contortus* single-worms and faecal larval cultures of sheep were used to amplify a partial β-tubulin isotype 1 encoding gene sequence harbouring the three most common mutation sites (at codon positions (P)200, (P)198 and (P)167), changes in which confer resistance to BZ drugs. The original primer sequences were described by Redman et al. (2015). Upon confirmation of successful amplification of a partial gene product of approximately 922 base-pairs (bp) in size (using a randomly selected, readily available genomic *H. contortus* DNA template) by conventional temperature gradient PCR, unique tags were developed to be used in the library creation step (Supplementary Table 2).

The conditions for the thermal cycling reactions were selected according to the manufacturer's guidelines (Thermo Scientific DreamTaq Green DNA Polymerase #EP0712) – a single cycle of initial denaturation at 95 °C for 2 min, followed by 30 cycles of denaturation at 95 °C for 30 s, annealing at 63 °C for 30 s and extension at 72 °C for 1 min. An additional one cycle of final extension at 72 °C for 10 min was included at the very end of the reaction. The final volume of samples was 15 μL. After the amplification step, samples were run on a 1% agarose gel and visualized by a BioRad Gel Doc™ system.

Amplified adult-worm DNA samples were pooled together in categories, according to the signal strengths of visualized bands on agarose gels (larval culture samples did not undergo the initial pooling step, but were subjected to the subsequent clean-up). These were then further subjected to a clean-up step using the AMPure XP magnetic beads, following the instructions detailed by the manufacturer (Beckman Coulter Inc.). The pooled pure partial β-tubulin locus sequences present in adult-worm and larval culture DNA samples were subjected to DNA quantification. Qubit dsDNA HS Assay Kit was employed to determine DNA concentrations in each sample, strictly following the guidelines issued by the manufacturer (Life Technologies). After this step, the remaining samples were further joined together (single-worm and larval culture samples were pooled separately) in equal concentrations forming five distinct batches and stored at −20 °C before sequencing. Pooled amplicon DNA samples were sent for sequencing to Uppsala Genome Center, Science for Life Laboratory, Dept. of Immunology, Genetics and Pathology, Uppsala University, BMC, Box 815, SE-752 37 UPPSALA and further sequenced using the PacBio RSII Technology Platform.

### 2.3 NGS data analysis

Sequencing data for both single-worms and larval culture pools was analysed using jMHC software (version 1.6.1624) (Stuglik et al., 2011). FASTA files for each sample pool were imported and samples were demultiplexed using unique nucleotide tag combinations. Sequencing errors were filtered out using jMHC and only sequences that were found in at least 3 reads were considered. The output file for single adult worms was analysed by manually enforcing strict criteria to filter out artefacts and obtain well-characterized DNA sequence variant(s) in each sample in three steps: (1) Sequences in samples were removed if they had less than three reads, (2) Assuming only up to two allele variants should be present in each single-worm sample, a sequence, whose coverage (i.e. number of reads) was lower than 50% of the highest coverage possessing sequence in that particular sample, was removed, (3) Samples containing multiple sequences with two or more possibilities of inferring binary allele combinations after steps (1) and (2) were removed. Sequence variants found in larval culture sample pools were also manually processed using these two criteria: (1) A sequence in a particular isolate sample was filtered out if its coverage was less than 3 reads, (2) Sequences present only in a single isolate sample and having a coverage of 3 or less were removed.

### 2.4 Phylogenetic analysis of partial β-tubulin sequences found in single-worm samples

The β-tubulin isotype 1 locus DNA sequences found in single-worm samples, which passed the filtering criteria, were kept and had their intron regions removed in order to construct a Bayesian phylogenetic tree using MrBayes software, version 3.2.6 (Ronquist et al., 2012). Settings used were as follows: Number of generations (Ngen; 200 mln), Number of chain swaps (Nswap; 10), Number of chains (Nchains; 10), Burnin-fraction (Burninfraq; 0.4), Number of substitution types (Nst; 6), and model for among-site variation (Rates; invgamma), whereas the other parameters were left at their default values. Phylogenetic relations between aligned sequences were then visualized using Dendroscope 3, version 3.5.9 (Huson and Linz, 2016).

### 2.5 Primer/probe development for droplet digital PCR

Primer sequences for ddPCR were first developed and verified by conventional PCR. CodonCode Aligner (version 7.1.1) was used to manually visualize and identify putative conserved regions, surrounding the mutation site of interest in the β-tubulin isotype 1 locus sequences from a variety of adult *H. contortus* isolates. A set of primers was then designed to anneal to those regions, whereas probes were developed separately. After successful tests were run with the primer sequences both *in silico*, using Primer3 (http://bioinfo.ut.ee/primer3/) and Sequence Manipulation Suite: PCR Primer Stats (www.bioinformatics.org/sms2/pcr_primer_stats.html), and *in vitro* using conventional PCR, two sets of primers and probes, designed specifically for ddPCR, were ordered (BioRad; Table 1).

**Table 1:** Primer and probe sets used for droplet digital PCR.

Primer-probe mix name:	Hc-β-tub – 200WT
Forward sequence:	TCGTGGAACCCTACAATGCT
Reverse sequence:	TCAAAGTGCGGAAGCAGATA
Probe sequence:	AACACCGATGAAACATTCTGTATTGAC
Fluorophore:	FAM™
Primer-probe mix name:	Hc-β-tub – 200MT
Forward sequence:	TCGTGGAACCCTACAATGCT
Reverse sequence:	TCAAAGTGCGGAAGCAGATA
Probe sequence:	AACACCGATGAAACATACTGTATTGAC
Fluorophore:	HEX™

### 2.6 ddPCR assay development

A ddPCR protocol was developed utilizing unique primer-probe sets and optimized for single-worm genomic (g)DNA samples with an intent on using this technique for the evaluation of the presence of mutant and/or wild-type alleles in extracted faecal larval culture gDNA isolates. The ddPCR reaction mixtures were assembled in 96-well plates at the total volume of 22 μL per well. A standard protocol provided by the manufacturer of the reagents was followed (ddPCR™ Supermix for Probes, catalog # 186–3010, BioRad). In short, a single reaction mixture was prepared by introducing 11 μL of 2x ddPCR Supermix for Probes, 1,1 μL of each 20x stock solution for primers/wild type probe (FAM™) and primers/mutant probe (HEX™) at the concentrations of 900 nM and 250 nM for primers and probes, respectively, and varying amounts of water and DNA template. Nanoliter size water-in-oil droplets were then generated using an automated droplet generator (along with DG32 Cartridges) and dispensed into a new 96-well plate. The plate containing the droplets was heat sealed with plastic foil (PX1 PCR Plate Sealer) and carefully transferred into the thermal cycler (MyCycler™ Thermal Cycler, BioRad). The thermal cycling conditions used for the PCR are readily described in the manufacturer's protocol. The temperature for the annealing step was chosen to be 58 °C, according to the results of the gradient ddPCR (data not shown).

After PCR amplification, the plate containing the droplets was loaded into the QX200 droplet reader (BioRad). As the automatic droplet generator is used to partition the 22 μL sample into approximately 20,000 water-in-oil droplets, the 2 channels for each fluorophore (FAM™ and HEX™) in the droplet reader are utilized to measure the amounts of fluorescent and non-fluorescent droplets. No-template-control (NTC), Wild-type only (WT) and Mutant only (MT) samples were included into the assays where necessary. Subsequent data was analysed using QuantaSoft software (version 1.7.4.0917). Thresholds, separating droplet clusters, were manually adjusted to obtain the best possible separation: Channel 1, which captures the fluorescence of the FAM™ dye molecule, attached to the wild type probe, had a threshold set at 7000 AU, whereas Channel 2, which respectively measures the fluorescence emitted by the HEX™ dye, attached to the mutant probe, at 5000 AU. A limit of detection (LoD) and fractional abundance (FA) precision identification in five wild-type (WT) and mutant (MT) mixtures (at 100/0, 75/25, 50/50, 25/75 and 0/100% ratios) tests (described by Elmahalawy et al., 2018) were carried out to evaluate the reliability of the designed assay.

### 2.7 Data comparison

Both NGS and ddPCR data on genomic DNA derived from larval cultures containing *H. contortus* were assembled and analysed for each sample. Sequences characterized by NGS as well as ddPCR were divided into wild-type (containing no mutation at P200) and mutant (containing an amino acid altering TTC→TAC transversion at P200) and were compared on the basis of the FA (in %):$\;FA\left(MT\right)=\;MT\left(\frac{copies}{\text{μL}}\right)÷\left(MT\left(\frac{copies}{\text{μL}}\right)+WT\left(\frac{copies}{\text{μL}}\right)\right)$ and *FA(WT) =* 100−FA(MT) for each allele between the two methods. MedCalc software (version 18.5) was utilized in the FA data processing and the visualization of the differences between the two methods (in the form of a Bland-Altman plot).

## 3 Results

### 3.1 Next generation PacBio RSII sequencing

Sequencing of the β-tubulin isotype 1 locus amplicons from single worm samples yielded 21984–27085 reads, 910–919 bp mean lengths of the products and the quality of insert varied between 98.92 and 99%. For amplicons from larval culture gDNA specimens, 31561–34677 reads, 892–898 mean bp lengths of the products and ∼99% quality of inserts were achieved. Upon processing of the sequences derived from both adult single-worm and larval culture gDNA, a total of 210 final samples (out of 223) successfully passed the imposed criteria (see *NGS data analysis* in Materials and methods). A total of 13 samples for single-worm specimens were removed due to not passing the set criteria.

For sequences obtained from single-worm samples, a total of 45 unique sequences were extracted and evaluated for genetic variation at a nucleotide level. A total of 219 nucleotide differences were observed between the sequences and the generated consensus, most of which were prominent in the intron regions. When the obtained exon sequences were translated *in silico* and compared, only three codons were shown to be polymorphic, i.e. P167, P198 and P200. Precisely 15 of the sequences were determined to harbour non-synonymous point mutations conferring resistance to BZ drugs (typical Phe→Tyr amino-acid substitutions at P167 and P200) and two of the sequences were identified to have previously unseen nucleotide mutations for *H. contortus* at the P198 (resulting in Glu→Leu and Glu→Thr substitutions). Further information about genetic relationships between the sequences at a nucleotide level was extracted by constructing a Bayesian phylogenetic tree (Supplementary Fig. 1). Although no immediate pattern of distribution or phylogeny was recognized, the different resistant genotypes were present in between 18 and 100% in the various samples from different countries such as Australia, Brazil, Canada, Ethiopia, Germany, Guadeloupe, Greece, Kenya and Sweden.

**Fig. 1:**
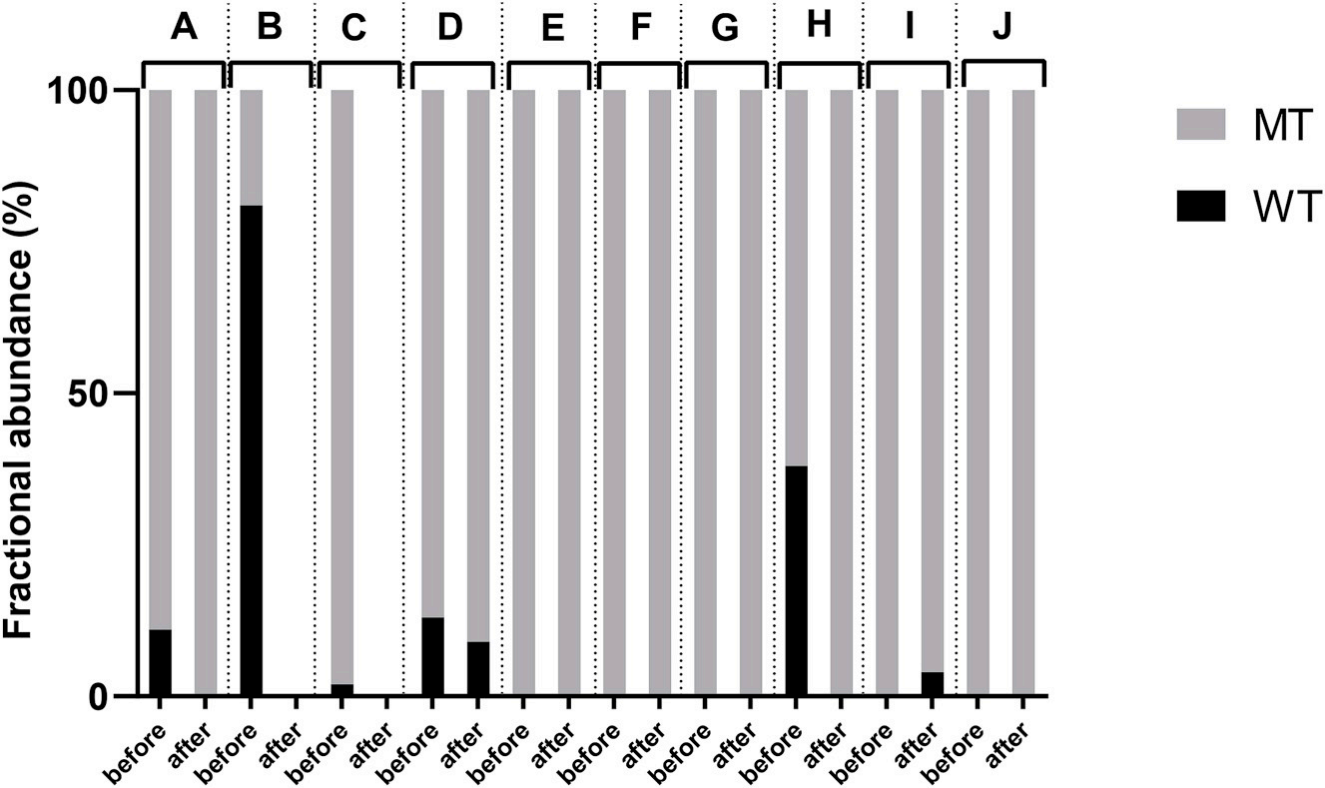
Relative fractional abundance (%) of *Haemonchus contortus* β-tubulin alleles (WT or MT at the P200) in larval cultures from different sheep farms (A–J) before and after BZ treatment. MT – fraction of mutant allele in the sample, WT – fraction of Wild-type allele in the sample.

For larval culture samples, obtained from sheep at different farms around Sweden pre- (before) and post- (after) treatment with the BZ drug, 32 unique β-tubulin isotype 1 locus sequences were retrieved. Among these, 22 had a typical F200Y (TAC) mutation conferring resistance to BZ drugs, while the other 10 were wild-type at P167, P198 and P200. The fractional abundance (FA) of wild-type and mutant *H. contortus* worms were further evaluated in pre- and post-treatment samples from ten different farms using the NGS data (referred to as A-J, Fig. 1). Farms A, D and H contrary to farm I show a decrease in the wild-type alleles post-treatment, whereas farms E, F, G and J show no change (despite G and J having reduced numbers of mutant sequence reads post-treatment) in the FA pattern pre- or post-treatment. Furthermore, post-treatment samples from farms B and C contained no amplifiable material, which was also indicated by the 100% reduction in the faecal egg count at these farms (conducted prior to this study; Supplementary Table 1).

### 3.2 Droplet digital PCR optimisation and reliability evaluation

**Fig. 2:**
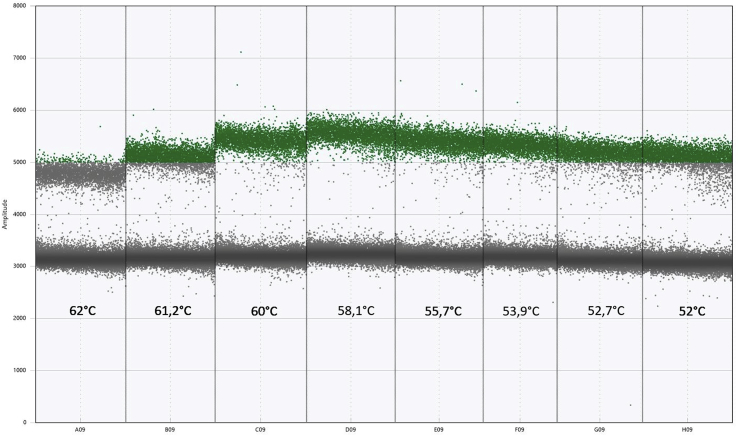
Optimal primer and probe annealing temperature determination for the ddPCR assay using a temperature gradient PCR. The highest amplitude of fluorescence and the best separation between bands, positive for wild-type (not shown) or mutant (green droplets) and negative (gray lowermost threshold), was observed in column D06, wherein annealing temperature was 58,1 °C. (For interpretation of the references to colour in this figure legend, the reader is referred to the Web version of this article.)

A gradient ddPCR was run to establish the most optimal temperature for the annealing step during the amplification. For both sets, wild-type probe/primers and mutant probe/primers, the clearest band separation, smallest observable droplet dispersion and highest amplitude of fluorescence was achieved at 58 °C (Column D in Fig. 2).

**Fig. 3:**
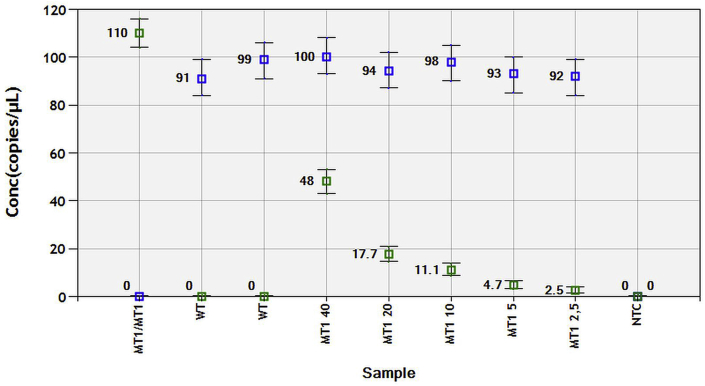
Limit of detection (LoD) for a mutant (MT) β-tubulin allele. A MT-labelled sample (MT1/MT1), containing mutant allele copies (green squares) was diluted (to 40, 20, 10, 5 and 2.5% of initial concentration) in the presence of a constant concentration of a wild (WT)-type allele copies (blue squares) (WT-MT1 2,5). Two negative controls were run as ‘WT’ (containing the WT only DNA) and non-treatment control (NTC). The ‘MT1 2,5’ well displayed a significantly different mutant DNA concentration from that of the WT well. Furthermore, the fractional abundance of wild-type DNA (blue rhombi) showed that in the well ‘MT1 2,5’ the fractional abundance of WT was 97.4%, meaning that the fraction of the MUT was 2.6%. Fractional abundance (of MT alleles) of 2.6% was then considered to be the limit of detection for mutant alleles. (For interpretation of the references to colour in this figure legend, the reader is referred to the Web version of this article.)

The LoD experiment displayed the capacity to identify a mixed sample, which contains MT alleles, if the concentration of those alleles was equal to or higher than 2.6% (Fig. 3). That is to say, a single MT DNA molecule can be identified out of roughly 38 total molecules in a sample. Therefore, a FA of 2.6% was considered to be a threshold value to help indicate a clear presence of mutant DNA.

**Fig. 4:**
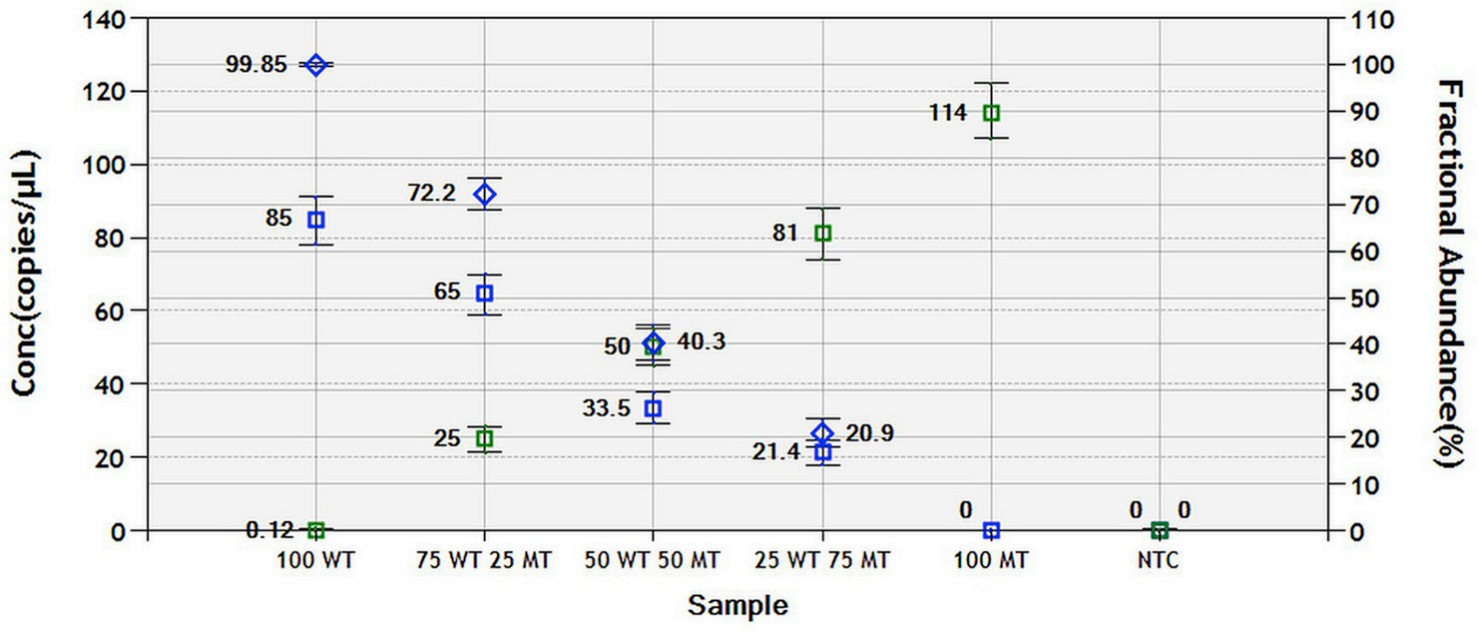
Fractional abundance (FA) precision (i.e. pilot) assay. Wild type (WT) and mutant (MT) samples of similar DNA concentrations were mixed at different ratios and the outcome evaluated through fractional abundance data. Samples were mixed in equal volumes, at ratios 100:0, 75:25, 50:50, 25:75 and 0:100. A clear and anticipated pattern can be observed, confirming the validity and robustness of the assay. Blue squares indicate the number of copies of WT alleles, whereas green squares - copies of MT alleles. Blue rhombi represent the fractional abundance of WT DNA in each sample (fractional abundance of MT = 100 – fractional abundance of WT). (For interpretation of the references to colour in this figure legend, the reader is referred to the Web version of this article.)

The FA precision (i.e. pilot) test demonstrated the variability in the FA between the samples to be up to ∼10% (Fig. 4), the most notable difference being observed when MT and WT DNA samples are mixed at equal ratios (1:1).

### 3.3 Data comparison between NGS and ddPCR

**Fig. 5:**
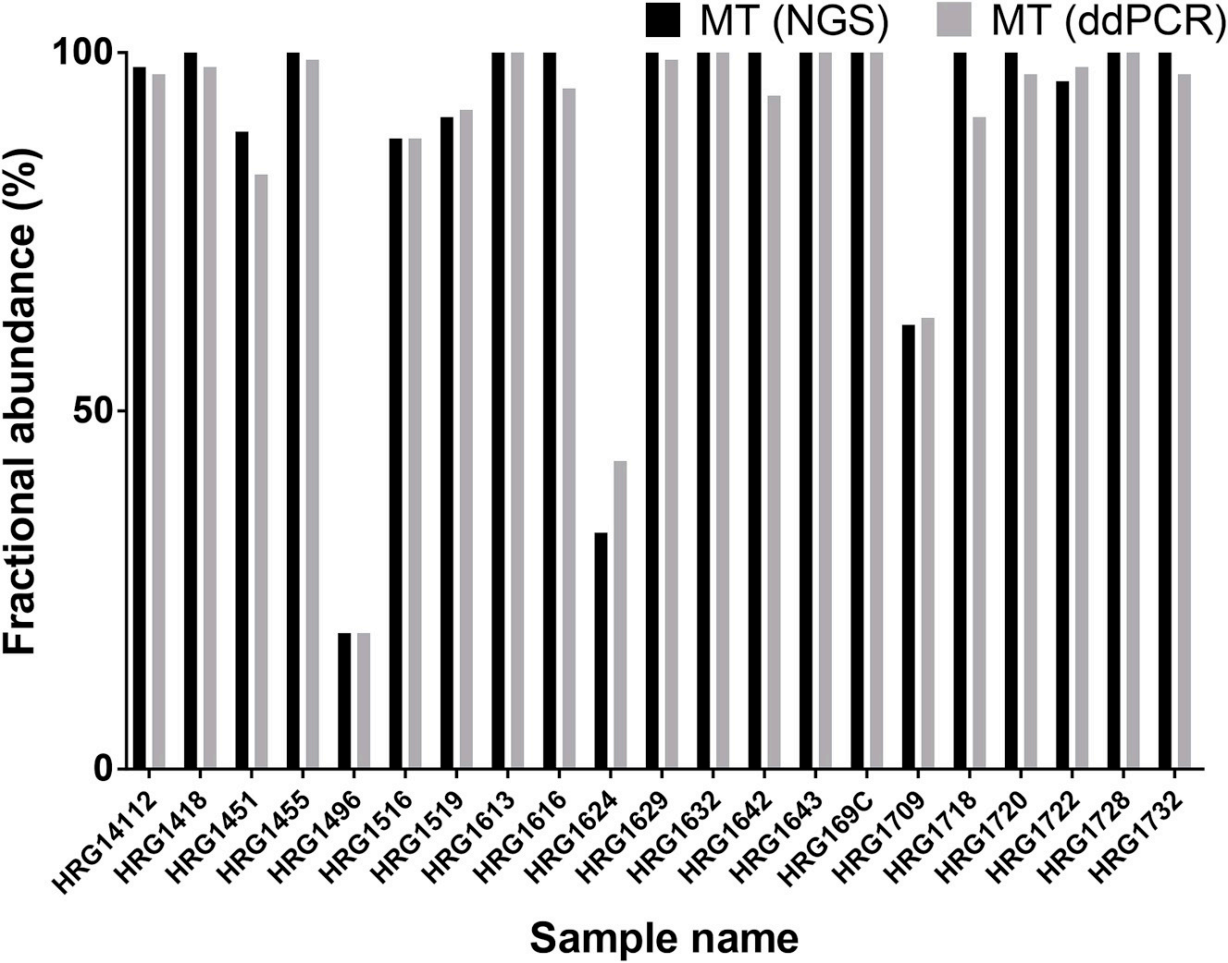
A comparison of identified mutant (MT) allele fractional abundances (in %) between next generation sequencing (NGS) and ddPCR techniques. Black bars – MT DNA fractional abundance (FA) in larval culture samples as determined by NGS, gray bars – MT DNA fractional abundance in the samples as determined by ddPCR.

**Fig. 6:**
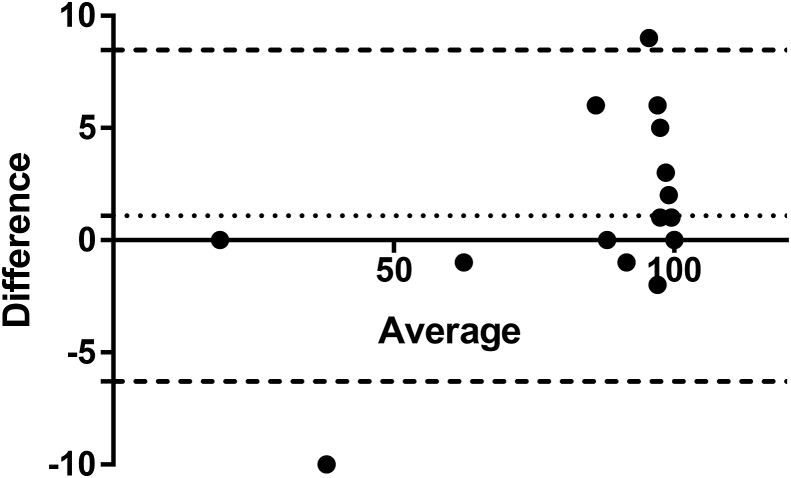
Bland-Altman [difference in fractional abundance (FA) plotted against mean values of the two measurement techniques] plot for highlighting differences in FA indices for MT sequences between ddPCR and NGS techniques. Round dots represent the differences between FA between the two methods, round-dotted line parallel to the x-axis (Y = 1.1) represents bias or mean value of the differences between measurements and two horizontal, rectangle-dotted lines (Y = 8.5 and −6.3) represent 95% limits of agreement.

The overall result of the comparison between the data extracted by these two methods yielded highly similar FA values (Fig. 5). Most data points (except for two – HRG1624 and HRG1718) in Fig. 6 fall in between 95% limits of agreement and generate a mean value of 1.1% difference in FA. The data from the two methods also displayed a linear correlation equal to r = 0.984 (95% confidence interval, CI = 0.971–0.995, P < 0.001). Wilcoxon matched-pairs signed rank test displayed no statistically significant difference between either the WT or MT FA values between the two methods (p = 0.1041).

## 4 Discussion

The relatively recent spread of BZ-resistant *H. contortus* into sheep flocks in Scandinavian countries, such as Denmark (Peña-Espinoza et al., 2014), Sweden (Höglund et al., 2009) and Norway (Domke et al., 2012) is truly worrying. In fact, most European countries are now compromised (Rose et al., 2015) as more cases of resistant strains come to light, leaving few options for European sheep farmers. It is theoretically possible, to at the very least attempt to halt the development and the establishment of resistance by treating affected individuals instead of the entire herd (Kenyon and Jackson, 2012). In order to achieve that, reliable and robust techniques must be presented, to ensure a quick, cheap and accurate screening of individual samples collected at farm level. Here, we propose droplet digital (dd)PCR as a novel platform for farm sample screening and resistance to BZ drugs identification by using β-tubulin isotype 1 gene locus as a marker in larval culture samples from animals infected with *H. contortus*.

Reports have been coming out over the last two decades heralding the worldwide distribution of drug resistant *H. contortus* strains (Waller and Chandrawathani, 2005; Kaplan and Vidyashankar, 2012; Rose et al., 2015). It was, therefore, important to look into the situation in regard to the BZ class drugs. From the 13 countries, wherein the single-worm samples were collected, in nine of them (Australia, Brazil, Canada, Ethiopia, Germany, Guadeloupe, Greece, Kenya and Sweden) mutant sequences were found. These findings essentially confirm results from several studies (e.g. Ghisi et al., 2007; Höglund et al., 2009; Mahieu et al., 2014; Barrere et al., 2013; Chaudhry et al., 2015; Ramünke et al., 2016; Zhang et al., 2016). Interestingly, single-worm samples from four locations (South Africa, Cambodia, Java and Argentina) showed only the presence of wild-type isolates. In the case of South Africa, it is highly suspicious that no mutants were detected, considering that it has been reported that the region is in dire situation in regard to anthelmintic resistance in sheep and goat parasites (Van Wyk et al., 1999). The same holds true for Argentina (Waller et al., 1996; Eddi et al., 1996) and South East Asian countries (Haryuningtyas, 2002). Thus, it is most likely that no resistant strains were found in these regions due to a limited pool of available, good-quality samples. What is more, the strains were isolated back in the early 2000s and might not be entirely representative of the present-time situations in these regions.

Of the total 45 identified partial β-tubulin isotype 1 locus sequences in single-worm samples, 24% (11) had the TTC/Phe → TAC/Tyr mutation at the P200, 9% (4) - TTC/Phe → TAC/Tyr at the P167 and 4% (2) - GAA/Glu → ? at the P198 (“?” being a different combination of nucleotides at this codon, leading to the incorporation of various amino acids). This pattern of mutation frequencies is in agreement with the previous work done by Barrere et al. (2013), Chaudhry et al. (2015), Ramünke et al. (2016) and Redman et al. (2015). Taken together, the data suggests that the first discovered F200Y (TAC) mutation in the β-tubulin isotype 1 gene of *H. contortus* is still the most relevant BZ resistance conferring mutation to this day. However, some discrepancy in mutation frequencies exists between different parts of the world, as a recent study conducted in China suggested the E198A (GCA) mutation in the β-tubulin isotype 1 gene to be the most widely abundant BZ resistance driving factor in *H. contortus* in 8 surveyed regions (Zhang et al., 2016). As it was previously described by several authors (Silvestre and Cabaret, 2002; Ghisi et al., 2007; Von Samson-Himmelstjerna et al., 2007), all adult worm DNA samples, in which we found mutant β-tubulin isotype 1 locus sequences, had only a single mutant codon per sequence, meaning no double or triple mutants for amino acids at P167, P198 or P200 were uncovered. Two of those β-tubulin isotype 1 locus DNA sequences contained previously undescribed, novel mutations at the P198 codon. Sequence 347, present in one of the samples isolated from Brazil, contained GAA→TTA (Glu→Leu) mutation, whereas Sequence 394, isolated from Sweden (Troell et al., 2006), harboured GAA→ACA (Glu→Thr) nucleotide sequence change. Both of these sequences were present in abundance in their respective samples and were not considered artefacts due to passing the filtering criteria. Furthermore, an analogue to Sequence 394, harbouring a Glu→Leu substitution at the P198, has been recently described but so far only for *T. circumcinta* (Redman et al., 2015 and Keegan et al., 2017). It is not entirely clear whether these genotypes confer increased resistance towards BZ class drugs in *H. contortus*. However, assuming they do, would indicate that a wider array of amino acid substitutions can be tolerated at the P198, while still maintaining the resistance status.

Out of the larval culture samples collected across the southern Sweden during the 2014–2017 period – 32 unique sequences were derived. Unexpectedly, as many as 22 (73%) of these were positive for the TTC→TAC transversion at the P200 in the β-tubulin isotype 1 gene locus, further confirming the postulation about the importance of the F200Y (TAC) substitution in BZ-resistance. Pre- and post-treatment (with albendazole) farm samples were evaluated for the presence of MT and/or WT alleles (Fig. 1). Only in farms B and C did the post-treatment samples contain no trace of amplifiable material, indicating a 100% effective treatment, which was also confirmed by FECRT data (Supplementary Table 1). In light of this, these samples were then excluded from the comparison between ddPCR and NGS test. The rest of the post-treatment samples across various farms displayed either a reduced WT allele frequencies or an unchanged presence pattern of MT sequences. This indicates that resistant strains are already wide-spread across the southern parts of Sweden, although, the initial FECRTs performed on these samples showed an efficient egg count reduction. Thus, the real situation seems to be much more complicated: the resistant strains were indeed somewhat reduced in number after treatment, but some continued to persist regardless of the treatment. This finding highlights the shortcomings of the FECRT method, mainly insensitivity (to detect resistant isolates), as reported before by others (Miller et al., 2006; Höglund et al., 2009).

The major objective of this study was to compare the allele frequency (i.e. fractional abundance (FA)) results obtained for the same larval cultures by NGS with those generated by ddPCR. For that reason, a novel ddPCR assay, encompassing a combination of unique primers with two (MT and WT) probes was developed. Although little was changed in the manufacturer's (BioRad) protocol, it was noticed early on that the addition of DNA restriction enzymes before (Supplementary Fig. 2) or during (not shown) droplet generation did not yield any beneficial effects in terms of amplification efficacy or droplet ‘rain’ reduction, contrary to what the manufacturers of the ddPCR machinery and ingredients claim (Rare Mutation Detection, Best Practices Guidelines; BioRad). In fact, diluting the sample so that an adequate amount of the droplets remain negative proved to be the best approach when attempting to maintain a good droplet distribution and clear band separation. Diluting samples could also be beneficial for improving the PCR as any PCR inhibitors would also be diluted.

Because both probes, being nearly identical in their sequences, compete for a single target site in a DNA molecule, some probe cross-reactivity was detected (Supplementary Fig. 2). This is observable in the form of a second, lower-amplitude fluorescence band in channels 1 and 2. In channel 1, fluorescence is produced with the help of the WT probe carrying a FAM™ fluorescent dye molecule, a second band can be seen just above the double-negative droplet threshold (around 5000 AU). In channel 2, where fluorescence is emitted with the help of the MT probe carrying a HEX™ fluorescence-producing molecule, a second band is seen at ∼4000 AU. Koch et al. (2016) observed a similar occurrence of the second band in channel 2 and concluded that it was likely due to the lower efficacy with which secondary dyes, such as HEX™ and VIC^®^ are detected. However, upon further discussion with BioRad technicians, it was suggested that these lower-specificity bands represent amplified DNA being bound to by the wrong probe (i.e. amplified mutant DNA binding WT probe and amplified WT DNA binding MT probe) and thus getting dispersed into the wrong channel. This is further confirmed by the decreased fluorescence intensity for these bands, as a single-base mismatch is expected to occur between the DNA and the probe in those circumstances. Taking this knowledge into account, manual thresholds for identifying positive droplets in both channels were established. We determined 7000 AU for channel 1 and 5000 AU for channel 2 to be sufficient and provide a somewhat good separation between non-specific and target bands.

Both the LoD and pilot test experiments displayed promising results. The LoD for some ddPCR assays has been reported to be as low as 0.01–0.04% of total mutant sequence abundance (Luo and Li, 2018). However, most of this type of research is focused on the detection of altered DNA in cancerous and/or pre-cancerous cells. Therefore, it is difficult to compare our results with those of other studies. Mutant larvae are multicellular and can obviously be present in high numbers in faecal cultures from infected animals. Thus, a threshold of 2.6% mutant sequence abundance for our LoD is a decent starting point that could potentially be further diminished through rigorous optimal condition testing if necessary. The pilot test, achieved by mixing MT and WT sequences at different ratios, showed a clear and reproducible pattern (Fig. 4). Although the FA values for WT sequences are slightly below the expected ones (blue rhombi), it is evident that the initial DNA copy number is slightly lower than that of the MT, thus resulting in minor deviations. We have subsequently ruled out contamination as a potential source for this variation, as out of 16 no-template control samples, run on different occasions, not a single positive droplet was recorded.

The final comparison between the FA data of the two alleles (WT and MT at the P200) obtained by NGS and ddPCR from paired larval cultures recovered both pre- and post-treatment with the BZ drug from different farms around the southern Sweden, showed impressive correlation (Fig. 5) (r = 0.988). The difference in abundance of alleles between the two methods, although varied to a maximum of 10% in some cases (HRG1624, HRG1718), showed very similar overall values. This was also confirmed by the Wilcoxon matched-pairs signed rank test, which indicated no statistically significant difference between the obtained data (p = 0.104). However, it appears that ddPCR is somewhat more sensitive of the two: being able to pick up more rare WT sequences (in samples HRG1418, HRG1455, HRG1629, HRG1720) as seen in Fig. 5. Nevertheless, it is arguable whether such small differences in abundances are of practical importance in terms of determining the resistance status of the entire worm population.

Incidence of drug resistant *H. contortus* has risen over time both in Europe and other parts of the world (Rose et al., 2015; Tsotetsi et al., 2013; Veríssimo et al., 2012; Falzon et al., 2013; Playford et al., 2014). This not only carries a threat to the health and welfare of grazing livestock, but it will also most likely result in monumental production losses. To combat the rise in AR in *H. contortus*, vigorous attempts at surveillance and the development of novel approaches for routine screening must be undertaken at regular intervals. To date, few studies have been published, wherein authors utilize ddPCR technology for the detection of parasites (e.g. Weerakoon et al., 2017; Koepfli et al., 2016) let alone those of veterinary importance. Here we propose a novel ddPCR approach to fractional allele abundance elucidation in samples containing mixed genotype *H. contortus* (resistant or susceptible to BZ drugs) larvae using β-tubulin isotype 1 gene locus as a genetic marker. We used the newly developed assay to further characterize BZ-drug treatment outcomes for 10 Swedish farms and compare these and additional results with those obtained with the NGS. Although promising data was acquired, more work needs to be done to implement the ddPCR technique in routine faecal sample examination.

## Conflicts of interest

The authors of this manuscript certify that they have NO affiliations with or involvement in any organization or entity with any financial interest, or non-financial interest in the subject matter discussed in this manuscript.

## Appendix A Supplementary data

Data Profile Data Profile

## References

[bib1] Barrere V., Falzon L.C., Shakya K.P., Menzies P.I., Peregrine A.S., Prichard R.K. (2013). Assessment of benzimidazole resistance in *Haemonchus contortus* in sheep flocks in Ontario, Canada: comparison of detection methods for drug resistance. Vet. Parasitol..

[bib2] Bauer C. (2001). Multispecific resistance of trichostrongyles to benzimidazoles in a goat herd in Germany. Dtsch. Tierärztliche Wochenschr. (DTW).

[bib3] Beech R.N., Prichard R.K., Scott M.E. (1994). Genetic variability of the β-tubulin genes in benzimidazole-susceptible and -resistant strains of *Haemonchus contortus*. Genetics.

[bib4] Besier R.B., Kahn L.P., Sargison N.D., Van Wyk J.A. (2016). The pathophysiology, ecology and epidemiology of *Haemonchus contortus* infection in small ruminants. Adv. Parasitol..

[bib5] Blouin M.S., Yowell C.A., Courtney C.H., Dame J.B. (1995). Host movement and the genetic structure of populations of parasitic nematodes. Genetics.

[bib6] Chaudhry U., Redman E.M., Raman M., Gilleard J.S. (2015). Genetic evidence for the spread of a benzimidazole resistance mutation across southern India from a single origin in the parasitic nematode *Haemonchus contortus*. Int. J. Parasitol..

[bib7] Coles G.C., Jackson F., Pomroy W.E., Prichard R.K., von Samson-Himmelstjerna G., Silvestre A., Taylor M.A., Vercruysse J. (2006). The detection of anthelmintic resistance in nematodes of veterinary importance. Vet. Parasitol..

[bib8] Cringoli G., Veneziano V., Rinaldi L., Sauvé C., Rubino R., Fedele V., Cabaret J. (2007). Resistance of trichostrongyles to benzimidazoles in Italy: a first report in a goat farm with multiple and repeated introductions. Parasitol. Res..

[bib9] Domke A.V., Chartier C., Gjerde B., Höglund J., Leine N., Vatn S., Stuen S. (2012). Prevalence of anthelmintic resistance in gastrointestinal nematodes of sheep and goats in Norway. Parasitol. Res..

[bib10] Eddi C., Caracostantogolo J., Peña M., Schapiro J., Marangunich L., Waller P.J., Hansen J.W. (1996). The prevalence of anthelmintic resistance in nematode parasites of sheep in Southern Latin America: Argentina. Vet. Parasitol..

[bib11] Elmahalawy S.T., Halvarsson P., Skarin M., Höglund J. (2018). Genetic variants in *dyf-7* validated by droplet digital PCR are not drivers for ivermectin resistance in *Haemonchus contortus*. Int. J. Parasitol.: Drugs and Drug Resistance.

[bib12] Falzon L.C., Menzies P.I., Shakya K.P., Jones-Bitton A., Vanleeuwen J., Avula J. (2013). Anthelmintic resistance in sheep flocks in Ontario, Canada. Vet. Parasitol..

[bib13] Ghisi M., Kaminsky R., Mäser P. (2007). Phenotyping and genotyping of *Haemonchus contortus* isolates reveals a new putative candidate mutation for benzimidazole resistance in nematodes. Vet. Parasitol..

[bib14] Haryuningtyas D.,.B. (2002). Methods of detection anthelmintic resistance in sheep and goats. WARTAZOA. Indonesian Bulletin of Animal and Veterinary Sciences.

[bib15] Hindson B.J., Ness K.D., Masquelier D.A., Belgrader P., Heredia N.J., Makarewicz A.J. (2011). High-throughput droplet digital PCR system for absolute quantitation of DNA copy number. Anal. Chem..

[bib16] Höglund J., Gustafsson K., Ljungström B.-L., Engström A., Donnan A., Skuce P. (2009). Anthelmintic resistance in Swedish sheep flocks based on a comparison of the results from the faecal egg count reduction test and resistant allele frequencies of the β-tubulin gene. Vet. Parasitol..

[bib17] Hong C., Hunt K.R., Coles G.C. (1996). Occurrence of anthelmintic resistant nematodes on sheep farms in England and goat farms in England and Wales. Vet. Rec..

[bib18] Hoste H., Sotiraki S., de Jesús Torres-Acosta J.F. (2011). Control of endoparasitic nematode infections in goats. Vet. Clin. Food Anim. Pract..

[bib19] Huson D., Linz S. (2016). Autumn algorithm - computation of hybridization networks for realistic phylogenetic trees. IEEE ACM Trans. Comput. Biol. Bioinf.

[bib20] Kaplan R.M., Vidyashankar A.N. (2012). An inconvenient truth: global worming and anthelmintic resistance. Vet. Parasitol..

[bib21] Keegan J.D., Good B., de Waal T., Fanning J., Keane O.M. (2017). Genetic basis of benzimidazole resistance in *Teladorsagia circumcincta* in Ireland. Ir. Vet. J..

[bib22] Kenyon F., Jackson F. (2012). Targeted flock/herd and individual ruminant treatment approaches. Vet. Parasitol..

[bib23] Koch H., Jeschke A., Becks L. (2016). Use of ddPCR in experimental evolution studies. Methods in Ecology and Evolution.

[bib24] Koepfli C., Nguitragool W., Hofmann N.E., Robinson L.J., Ome-Kaius M., Sattabongkot J. (2016). Sensitive and accurate quantification of human malaria parasites using droplet digital PCR (ddPCR). Sci. Rep..

[bib25] Kotze A.C., Prichard R.K. (2016). Anthelmintic resistance in *Haemonchus contortus*: history, mechanisms and diagnosis. Adv. Parasitol..

[bib26] Lane J., Jubb T., Shephard R., Webb-Ware J., Fordyce G. (2015). MLA Final Report: Priority List of Endemic Diseases for the Red Meat Industries. http://www.wormboss.com.au/files/pages/worms/roundworms/the-cost-of-roundworms/B.AHE.0010_%20Final_Report.pdf.

[bib27] Levecke B., Dobson R.J., Speybroeck N., Vercruysse J., Charlier J. (2012). Novel insights in the faecal egg count reduction test for monitoring drug efficacy against gastrointestinal nematodes of veterinary importance. Vet. Parasitol..

[bib28] Luo Y., Li Y. (2018). Detection of KRAS mutation in colorectal cancer patients' cfDNA with droplet digital PCR. Chin. J. Biotechnol..

[bib29] Mahieu M., Ferré B., Madassamy M., Mandonnet N. (2014). Fifteen years later, anthelmintic resistances have dramatically spread over goat farms in Guadeloupe. Vet. Parasitol..

[bib30] McLeod R.S., Sani R.A., Gray G.D., Baker R.L. (2004). Economic impact of worm infections in small ruminants in South East Asia, India and Australia. Worm Control of Small Ruminants in Tropical Asia.

[bib31] Mickiewicz M., Czopowicz M., Górski P., Kaba J. (2017). The first reported case of resistance of gastrointestinal nematodes to benzimidazole anthelmintic in goats in Poland. Annals of Parasitology.

[bib32] Miller C., Waghorn T., Leathwick D., Gilmour M. (2006). How repeatable is a faecal egg count reduction test?. New Zealand Veterinary Journal.

[bib33] Nieuwhof G.J., Bishop S.C. (2005). Costs of the major endemic diseases of sheep in Great Britain and the potential benefits of reduction in disease impact. Anim. Sci..

[bib34] Paraud C., Kulo A., Pors I., Chartier C. (2009). Resistance of goat nematodes to multiple anthelmintics on a farm in France. Vet. Rec..

[bib35] Peña-Espinoza M., Thamsborg S.M., Demeler J., Enemark H.L. (2014). Field efficacy of four anthelmintics and confirmation of drug-resistant nematodes by controlled efficacy test and pyrosequencing on a sheep and goat farm in Denmark. Vet. Parasitol..

[bib36] Playford M., Smith A., Love S., Besier R., Kluver P., Bailey J. (2014). Prevalence and severity of anthelmintic resistance in ovine gastrointestinal nematodes in Australia (2009-2012). Aust. Vet. J..

[bib37] Ramünke S., Melville L., Rinaldi L., Hertzberg H., de Waal T., von Samson-Himmelstjerna G. (2016). Benzimidazole resistance survey for *Haemonchus, Teladorsagia* and *Trichostrongylus* in three European countries using pyrosequencing including the development of new assays for *Trichostrongylus*. Int. J. Parasitol.: Drugs and Drug Resistance.

[bib38] Redman E., Whitelaw F., Tait A., Burgess C., Bartley Y., Skuce P.J. (2015). The emergence of resistance to the benzimidazole anthlemintics in parasitic nematodes of livestock is characterised by multiple independent hard and soft selective sweeps. PLoS Neglected Trop. Dis..

[bib39] Requejo-Fernández J.A., Martínez A., Meana A., Rojo-Vázquez F.A., Osoro K., Ortega-Mora L.M. (1997). Anthelmintic resistance in nematode parasites from goats in Spain. Vet. Parasitol..

[bib40] Ronquist F., Teslenko M., van der Mark P., Ayres D.L., Darling A., Höhna S. (2012). MrBayes 3.2: efficient Bayesian phylogenetic inference and model choice across a large model space. Syst. Biol..

[bib41] Rose H., Rinaldi L., Bosco A., Mavrot F., de Waal T., Skuce P. (2015). Widespread anthelmintic resistance in European farmed ruminants: a systematic review. Vet. Rec..

[bib42] Schnyder M., Torgerson P.R., Schönmann M., Kohler L., Hertzberg H. (2005). Multiple anthelmintic resistance in *Haemonchus contortus* isolated from South African Boer goats in Switzerland. Vet. Parasitol..

[bib43] Silvestre A., Cabaret J. (2002). Mutation in position 167 of isotype 1 β-tubulin gene of Trichostrongylid nematodes: role in benzimidazole resistance?. Mol. Biochem. Parasitol..

[bib44] Stuglik M.T., Radwan J., Babik W. (2011). jMHC: software assistant for multilocus genotyping of gene families using next-generation amplicon sequencing. Molecular Ecology Resources.

[bib45] Sutherland I.A., Leathwick D.M. (2011). Anthelmintic resistance in nematode parasites of cattle: a global issue?. Trends Parasitol..

[bib47] Troell K., Engström A., Morrison D.A., Mattsson J.G., Höglund J. (2006). Global patterns reveal strong population structure in *Haemonchus contortus*, a nematode parasite of domesticated ruminants. Int. J. Parasitol..

[bib48] Tsotetsi A.M., Njiro S., Katsande T.C., Moyo G., Baloyi F., Mpofu J. (2013). Prevalence of gastrointestinal helminths and anthelmintic resistance on small-scale farms in Gauteng Province, South Africa. Trop. Anim. Health Prod..

[bib49] Van den Brom R., Moll L., Borgsteede F.H.M., Van Doorn D.C.K., Lievaart-Peterson K., Dercksen D.P., Vellema P. (2013). Multiple anthelmintic resistance of *Haemonchus contortus*, including a case of moxidectin resistance, in a Dutch sheep flock. Vet. Rec..

[bib50] Van Wyk J.A., Stenson M.O., Van der Merwe J.S., Vorster R.J., Viljoen P.G. (1999). Anthelmintic resistance in South Africa: surveys indicate an extremely serious situation in sheep and goat farming. Onderstepoort J. Vet. Res..

[bib51] Veríssimo C.J., Niciura S.C.M., Alberti A.L.L., Rodrigues C.F.C., Barbosa C.M.P., Chiebao D.P. (2012). Multidrug and multispecies resistance in sheep flocks from São Paulo state, Brazil. Vet. Parasitol..

[bib52] Von Samson-Himmelstjerna G., Blackhall W.J., McCarthy J.S., Skuce P.J. (2007). Single nucleotide polymorphism (SNP) markers for benzimidazole resistance in veterinary nematodes. Parasitology.

[bib53] Von Samson-Himmelstjerna G., Walsh T.K., Donnan A.A., Carrière S., Jackson F., Skuce P.J. (2009). Molecular detection of benzimidazole resistance in *Haemonchus contortus* using real-time PCR and pyrosequencing. Parasitology.

[bib54] Waller P.J., Chandrawathani P. (2005). *Haemonchus contortus*: parasite problem No. 1 from Tropics-Polar Circle. Problems and prospects for control based on epidemiology. Trop. Biomed..

[bib55] Waller P.J., Echevarria F., Eddi C., Maciel S., Nari A., Hansen J.W. (1996). The prevalence of anthelmintic resistance in nematode parasites of sheep in Southern Latin America: general overview. Vet. Parasitol..

[bib56] Weerakoon K.G., Gordon C.A., Williams G.M., Cai P., Gobert G.N., Olveda R.M. (2017). Droplet digital PCR diagnosis of human schistosomiasis: parasite cell-free DNA detection in diverse clinical samples. J. Infect. Dis..

[bib57] Zhang Z., Gasser R.B., Yang X., Yin F., Zhao G., Bao M. (2016). Two benzimidazole resistance-associated SNPs in the isotype-1 β-tubulin gene predominate in *Haemonchus contortus* populations from eight regions in China. International Journal for Parasitology. Drugs and Drug Resistance.

