# Supplementary material for: Exploring benzimidazole resistance in Haemonchus contortus by next generation sequencing and droplet digital PCR
Source: Int J Parasitol Drugs Drug Resist. 2018 Sep 19;8(3):411–9. doi: 10.1016/j.ijpddr.2018.09.003 (PMC6159336; doi:10.1016/j.ijpddr.2018.09.003)

**Supplementary File**

Table 1. Nematode eggs per gram (EPG) counts in faeces performed for sheep on Farms A-M. For farms K-M only a single sample was retrieved either pre- (before) or post- (after) treatment. The proportion of eggs belonging to *H. contortus* was expressed in %

| **Farm name** | **Sample name** | **Treatment** | **EPG** | **% *H. contortus*** |
| --- | --- | --- | --- | --- |
| Farm A | HRG1451 | before | 4023 | 85 |
|  | HRG1455 | after | 0 | 0 |
| Farm B | HRG1496 | before | 423 | 40 |
|  | HRG14101 | after | 0 | 0 |
| Farm C | HRG14112 | before | 641 | 68 |
|  | HRG14118 | after | 10 | 0 |
| Farm D | HRG1516 | before | 1407 | 91 |
|  | HRG1519 | after | 70 | 100 |
| Farm E | HRG169C | before | 257 | 90 |
|  | HRG1613 | after | 20 | 100 |
| Farm F | HRG1629 | before | 1532 | 83 |
|  | HRG1632 | after | 500 | 48 |
| Farm G | HRG1642 | before | 1980 | 78 |
|  | HRG1643 | after | 0 | 0 |
| Farm H | HRG1709 | before | 5577 | 81 |
|  | HRG1718 | after | 35 | 25 |
| Farm I | HRG1720 | before | 653 | 60 |
|  | HRG1722 | after | 67 | 30 |
| Farm J | HRG1728 | before | 567 | 77 |
|  | HRG1732 | after | 0 | 0 |
| Farm K | HRG1418 | after | 195 | 90 |
| Farm L | HRG1616 | before | 1433 | 87 |
| Farm M | HRG1624 | before | 370 | 45 |

Table 2. Primer tags used in partial β-tubulin library creation

| Primer name | tag number | 10 bp tag |
| --- | --- | --- |
| Hc37R | tag_001 | GACACGATCG |
| Hc37R | tag_004 | GACATAGTCC |
| Hc37R | tag_005 | GACATGACTG |
| Hc37R | tag_006 | GACGATCAGC |
| Hc37R | tag_007 | GACGTGCTCG |
| Hc37R | tag_008 | GACTATAGCC |
| Hc37R | tag_009 | GACTATGTGG |
| Hc37R | tag_010 | GACTCAGAGC |
| Hc37R | tag_011 | GACTCTCACG |
| Hc37R | tag_012 | GACTGCTACC |
| Hc37R | tag_013 | GAGACAGTGG |
| Hc37R | tag_014 | GAGACATCTC |
| Hc37F | tag_001 | GACACGATCG |
| Hc37F | tag_004 | GACATAGTCC |
| Hc37F | tag_005 | GACATGACTG |
| Hc37F | tag_006 | GACGATCAGC |
| Hc37F | tag_007 | GACGTGCTCG |
| Hc37F | tag_008 | GACTATAGCC |
| Hc37F | tag_009 | GACTATGTGG |
| Hc37F | tag_010 | GACTCAGAGC |
| Hc37F | tag_011 | GACTCTCACG |
| Hc37F | tag_012 | GACTGCTACC |
| Hc37F | tag_013 | GAGACAGTGG |
| Hc37F | tag_014 | GAGACATCTC |

Figure 1. Bayesian phylogenetic tree, assembled from exon sequences discovered in single-worm *Haemonchus contortus* samples from different countries around the world. Exon sequences for β-tubulin in *H. placei* were used as an outgroup. Numbers above the branches represent the probability (bootstrap) values. Black branches represent WT sequences for 200th, 198th and 167th codons in β-tubulin gene, red branches – MT sequences for 200th codon (TTC→TAC), blue branches – MT sequences for 167th codon (TTC→TAC), purple – MT sequences for 198th codon (GAA→TTA and GAA→ACA). Each branch represents an individual sequence, which has a denoted sequence (seq) number and a country of origin*.*


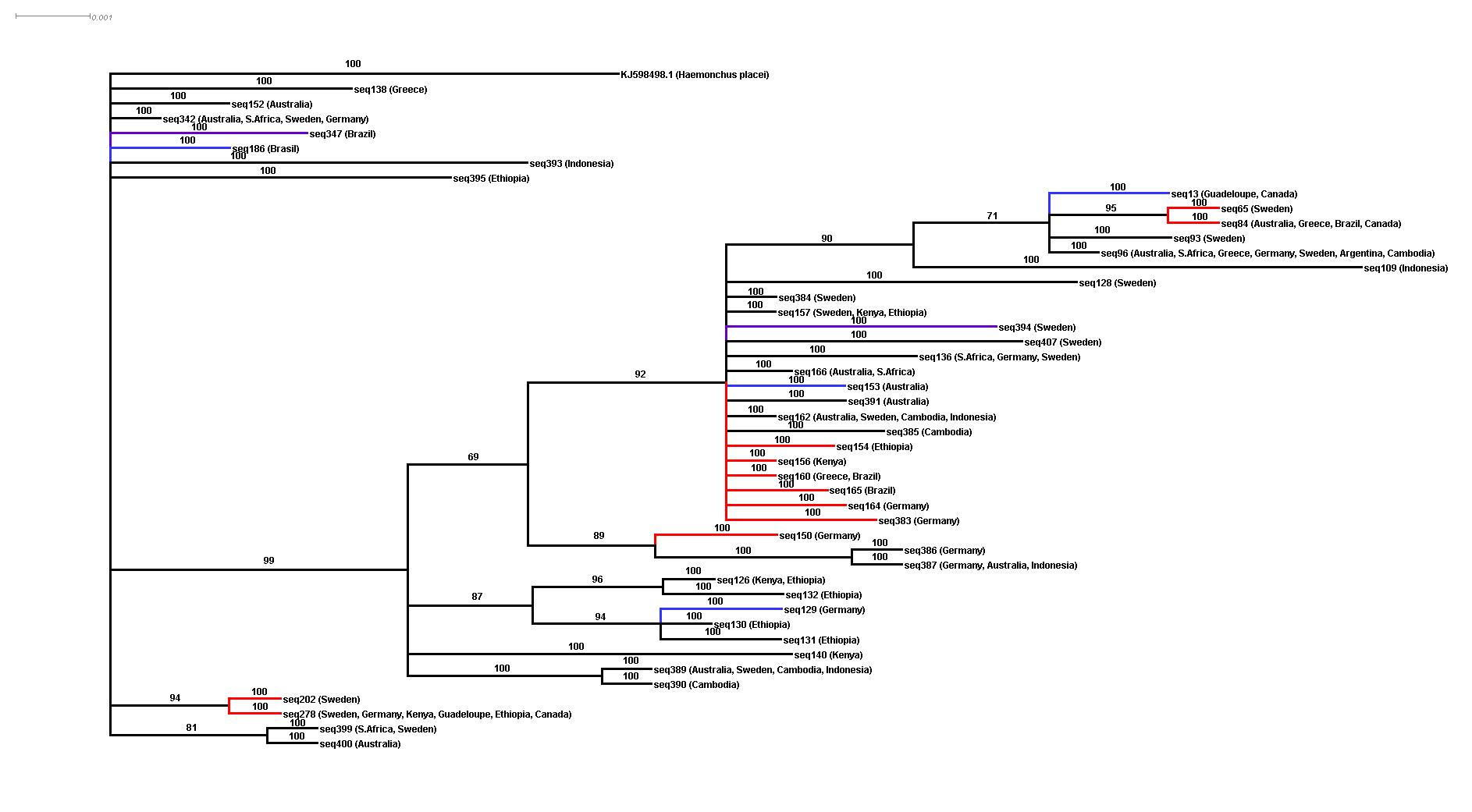


Figure 2. Enzymatic DNA digestion before droplet generation. DNA was incubated in an assembled reaction mix at 37^o^C with BglII restriction enzyme for different periods of time, 60 minutes (60 min.), 30 minutes (30 min.) and 0 minutes (0 min.; no incubation).


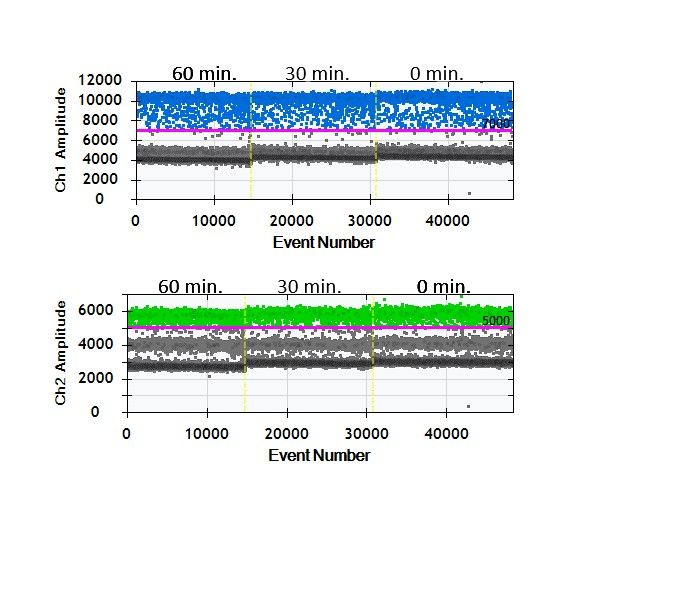

Supplement: Supplementary file [file mmc2.docx]
